# Outcome of Relapsed or Refractory *FLT3*-Mutated Acute Myeloid Leukemia before Second-Generation FLT3 Tyrosine Kinase Inhibitors: A Toulouse–Bordeaux DATAML Registry Study

**DOI:** 10.3390/cancers12040773

**Published:** 2020-03-25

**Authors:** Sarah Bertoli, Pierre-Yves Dumas, Emilie Bérard, Laetitia Largeaud, Audrey Bidet, Eric Delabesse, Suzanne Tavitian, Noémie Gadaud, Thibaut Leguay, Harmony Leroy, Jean-Baptiste Rieu, Jean-Philippe Vial, François Vergez, Nicolas Lechevalier, Isabelle Luquet, Emilie Klein, Audrey Sarry, Anne-Charlotte De Grande, Christian Récher, Arnaud Pigneux

**Affiliations:** 1 Service d’Hématologie, Centre Hospitalier Universitaire de Toulouse, Institut Universitaire du Cancer de Toulouse-Oncopole, 31059 Toulouse, France; tavitian.suzanne@iuct-oncopole.fr (S.T.); gadaud.noemie@iuct-oncopole.fr (N.G.); sarry.audrey@iuct-oncopole.fr (A.S.); recher.christian@iuct-oncopole.fr (C.R.); 2 University Toulouse III Paul Sabatier, 31000 Toulouse, France; largeaud.laetitia@iuct-oncopole.fr (L.L.); delabesse.eric@iuct-oncopole.fr (E.D.); vergez.francois@iuct-oncopole.fr (F.V.); 3 Cancer Research Center of Toulouse, UMR1037 INSERM, ERL5294 CNRS, 31000 Toulouse, France; 4 CHU Bordeaux, Service d’Hématologie Clinique et de Thérapie Cellulaire, F-33000 Bordeaux, France; pierre-yves.dumas@u-bordeaux.fr (P.-Y.D.); thibaut.leguay@chu-bordeaux.fr (T.L.); leroy.harmony@gmail.com (H.L.); anne-charlotte.de-grande@chu-bordeaux.fr (A.-C.D.G.); arnaud.pigneux@chu-bordeaux.fr (A.P.); 5 Université de Bordeaux, 33000 Bordeaux, France; 6 Institut National de la Santé et de la Recherche Médicale, U1035, 33000 Bordeaux, France; 7 Service d’Epidémiologie, Centre Hospitalier Universitaire de Toulouse, 31000 Toulouse, France; emilie.berard@univ-tlse3.fr; 8 UMR 1027, INSERM-Université de Toulouse III, 31000 Toulouse, France; 9 Laboratoire d’Hématologie, Centre Hospitalier Universitaire de Toulouse, Institut Universitaire du Cancer de Toulouse-Oncopole, 31059 Toulouse, France; rieu.jeanbaptiste@iuct-oncopole.fr (J.-B.R.); luquet.isabelle@iuct-oncopole.fr (I.L.); 10 CHU Bordeaux, Laboratoire d’Hématologie Biologique, F-33000 Bordeaux, France; audrey.bidet@chu-bordeaux.fr (A.B.); jean-philippe.vial@chu-bordeaux.fr (J.-P.V.); nicolas.lechevalier@chu-bordeaux.fr (N.L.); emilie.klein@chu-bordeaux.fr (E.K.)

**Keywords:** acute myeloid leukemia, *FLT3*-ITD mutation, *FLT3*-TKD mutation, primary induction failure, refractory, relapse, tyrosine kinase inhibitors, gilteritinib

## Abstract

A recent phase 3 trial showed that the outcome of patients with relapsed/refractory (R/R) *FLT3*-mutated acute myeloid leukemia (AML) improved with gilteritinib, a single-agent second-generation FLT3 tyrosine kinase inhibitor (TKI), compared with standard of care. In this trial, the response rate with standard therapy was particularly low. We retrospectively assessed the characteristics and outcome of patients with R/R *FLT3*-mutated AML included in the Toulouse–Bordeaux DATAML registry. Among 347 patients who received FLT3 TKI-free intensive chemotherapy as first-line treatment, 174 patients were refractory (*n* = 48, 27.6%) or relapsed (*n* = 126, 72.4%). Salvage treatments consisted of intensive chemotherapy (*n* = 99, 56.9%), azacitidine or low-dose cytarabine (*n* = 9, 5.1%), other low-intensity treatments (*n* = 17, 9.8%), immediate allogeneic stem cell transplantation (*n* = 4, 2.3%) or best supportive care only (*n* = 45, 25.9%). Among the 114 patients who previously received FLT3 TKI-free intensive chemotherapy as first-line treatment (refractory, *n* = 32, 28.1%; relapsed, *n* = 82, 71.9%), the rate of CR (complete remission) or CRi (complete remission with incomplete hematologic recovery) after high- or low-intensity salvage treatment was 50.0%, with a bridge to transplant in 34.2% (*n* = 39) of cases. The median overall survival (OS) was 8.2 months (interquartile range, 3.0–32); 1-, 3- and 5-year OS rates were 36.0% (95%CI: 27–45), 24.7% (95%CI: 1–33) and 19.7% (95%CI: 1–28), respectively. In this real-word study, although response rate appeared higher than the controlled arm of the ADMIRAL trial, the outcome of patients with R/R *FLT3*-mutated AML remains very poor with standard salvage therapy.

## 1. Introduction

FMS-like tyrosine kinase 3 (FLT3) is a class III receptor tyrosine kinase expressed in early hematopoietic stem and progenitor cells that regulates their proliferation and differentiation [1]. *FLT3*-activating mutations occur in approximately 30% of patients with acute myeloid leukemia (AML) and, as such, are among the most frequent mutations found in AML [2], either as in-frame internal tandem duplications (ITD) within the juxtamembrane region or as missense point mutations in the tyrosine kinase domain (TKD) [3,4]. *FLT3*-ITD (but not *FLT3*-TKD) mutations confer a poor prognosis in AML, especially when *NPM1* is not co-mutated and the allelic *FLT3*-ITD/wild-type ratio is high; such mutations are usually conserved at relapse and have therefore emerged as a relevant therapeutic target [5,6]. FLT3 tyrosine kinase inhibitors have been developed for several years, and the type I FLT3 inhibitor (i.e., that has activity against *FLT3*-ITD and TKD mutations) midostaurin was approved in 2017 for the treatment of newly diagnosed *FLT3*-mutated AML, in combination with intensive chemotherapy [7]. Furthermore, several second-generation FLT3 inhibitors, such as quizartinib, gilteritinib and crenolanib, have demonstrated single-agent activity that can lead to complete or near-complete remission [8,9,10].

The ADMIRAL phase 3 trial, designed for relapsed/refractory (R/R) *FLT3*-mutated AML patients, recently demonstrated the superiority of gilteritinib as single agent over the control treatment arm [8], which was determined by investigators prior to 2:1 randomization between mitoxantrone, etoposide, cytarabine (MEC), fludarabine, cytarabine, granulocyte colony-stimulating factor, idarubicin (FLAG-IDA), azacitidine (AZA) or low-dose cytarabine (LDAC). These regimens are recognized as acceptable salvage regimens in this situation, although other combinations based on intermediate- or high-dose cytarabine or even single-agent cytarabine are also widely used [11]. In the ADMIRAL trial, overall survival (OS) was significantly improved in the gilteritinib arm compared to the control arm with a hazard ratio (HR) of 0.64 (95%CI: 0.49–0.83; *p* < 0.001). The median OS was 9.3 months in the gilteritinib arm and 5.6 months in the control arm. The complete remission and complete remission with incomplete hematologic recovery rates were 21.1% and 25.5% in the gilteritinib arm vs. 10.5% and 4.8% in the standard arm [8].

The aim of our study was to describe the characteristics, treatments and outcome of R/R *FLT3*-mutated AML patients treated in a routine setting before the approval of second-generation FLT3 inhibitors in this indication.

## 2. Materials and Methods

### 2.1. Patients and Treatment

This retrospective study included all patients with newly diagnosed AML according to 2016 WHO classification [12], excluding acute promyelocytic leukemia, between January 1, 2000 and December 31, 2017. All patients were registered in the Toulouse–Bordeaux DATAML registry. Patients were included in the current study if they were ≥18 years of age, treated by first-line intensive chemotherapy [13], with an *FLT3*-ITD or *FLT3*-TKD mutation and an ITD/wild-type (wt) ratio greater than 0.03. Written informed consent was obtained in accordance with the Declaration of Helsinki, allowing for the collection of clinical data in the anonymized database. Cytogenetic risk classification was defined according to the Medical Research Council classification [14]. Main salvage regimens used in both centers were based on single-agent cytarabine (high-dose cytarabine, 3 g/m^2^/12 h, d1–4; intermediate dose-cytarabine, 1 g–1.5 g/m^2^/12 h, d1–4 or 1 g/m^2^/d, d1–5), combination of an anthracycline plus cytarabine (daunorubicin 60 mg/m^2^/d, d1–3 or idarubicin 12 mg/m^2^/d, d1–3 or amsacrine 200 mg/m^2^/d, d1–3 + cytarabine 1.5–3 g/m^2^/12 h, d1–4), or the FLAG-IDA regimen (fludarabine 30 mg/m^2^/d, d1–5 + cytarabine 2 g/m^2^/d, d1–5 + idarubicin 10 mg/m^2^/d, d1–3 + G-CSF 5 µg/kg/d, d1–5). The choice between these different options was made on a case-by-case basis depending on the patient’s performance status, previous treatment history and time to relapse. Gemtuzumab ozogamycin was occasionally used. Sorafenib was also used off-label after preliminary results showing efficacy in *FLT3*-ITD R/R AML patients [15]. Bone marrow assessment was performed for patients treated by intensive chemotherapy after peripheral recovery or in case of delayed recovery, between days 35 and 45. Response to treatment, relapse-free survival (RFS), event-free survival (EFS), cumulative incidence of relapse (CIR) and overall survival (OS) were defined according to the European LeukemiaNet 2017 criteria [5]. For R/R AML, OS and EFS were measured from the date of relapse or the date of failure; RFS and CIR were measured from complete remission obtained after salvage therapy. Primary refractory AML was defined as a failure to achieve complete remission (CR) or CR with incomplete hematologic recovery (CRi) after one or two courses of induction chemotherapy; relapse was defined as bone marrow blasts ≥ 5%, reappearance of blasts in the blood or development of extramedullary disease [5].

### 2.2. Statistical Analysis

We described the patients’ characteristics at diagnosis and at relapse using numbers and frequencies for qualitative data and using the median, interquartile range (IQR) and range (minimum–maximum) for quantitative data. For survival analyses of RFS, EFS and OS, Kaplan–Meier survival curves were drawn and described using median, IQR and survival at 1, 3 and 5 years. For relapse, cumulative incidence functions were drawn (since nonrelapse mortality was treated as a competing event) and described using CIR at 1, 3 and 5 years. Hazard ratios (HR) and 95% confidence intervals (95%CI) were assessed using a standard Cox model for RFS, EFS and OS and using a proportional subdistribution hazard model (an extension of the Cox model) for competing risks for CIR [16]. For the rate of CR or CRi, odds ratios (OR) and 95% confidence intervals (95%CI) were assessed using a standard logistic regression model. Multivariate analyses, for newly diagnosed *FLT3*-ITD and *FLT3*-TKD AML patients initially included all potential risk factors (age, performance status (ECOG), AML status (de novo or secondary AML), gender, white blood cells, cytogenetics risk, *NPM1* co-mutation status and allogeneic hematopoietic stem cell transplantation (HSCT; only for EFS, RFS, CIR and OS)) associated with endpoints with a *p*-value less than 0.20 in univariate analyses. A stepwise regression was then used to assess variables that were significantly and independently associated with the endpoints (*p*-value < 0.05). The proportional hazard assumption was tested for each covariate of the Cox model using log–log plot method curves and was always supported. When the linear hypothesis was not supported, continuous potential confounding factors were transformed into ordered data. Interactions between variables that were significantly and independently associated with endpoints were tested in final models. None were significant. Allogeneic HSCT was evaluated as a time-dependent covariate. All reported *p*-values were two-sided, and the significance threshold was <0.05. Statistical analyses were performed using STATA version 14.2 (STATA Corp., College Station, TX, USA).

## 3. Results

### 3.1. Study Population

Out of 3290 newly diagnosed AML patients included in the DATAML registry between 2000 and 2017, 1453 did not have a recorded status for *FLT3* mutation and 364 were not selected to receive intensive chemotherapy as a first-line treatment. A total of 347 patients with *FLT3*-ITD (*n* = 317) or *FLT3*-TKD (*n* = 39) mutated AML fulfilled the inclusion criteria (Figure S1). Their characteristics are presented in Table 1. One hundred fifty-three patients (44.1%) were 60 years of age or older. There were 306 (88.4%) de novo AML. Extramedullary involvement and leukostasis were observed in 132 (42.7%) and 53 (15.5%) patients, respectively. The median white blood cell count (WBC) was 52.6 × 10^9^/L (IQR: 20.6–117.8). In *FLT3*-ITD-mutated patients with documented allelic ratio, 67 out of 141 (47.5%) had an ITD/wt ratio greater than 0.5. The vast majority of patients (*N* = 318, 91.6%) had an intermediate cytogenetic risk (normal karyotype: *N* = 255/311 (82%)). Two hundred fourteen patients out of 326 (65.6%) had an *NPM1* co-mutation, and 52 out of 143 patients had a *DNMT3A* co-mutation (36.4%).

### 3.2. First-Line Treatment and Outcome

Treatment regimens for induction chemotherapy based on anthracyclines and cytarabine are presented in Table 1. One hundred fifty-one patients (43.9%) received hydroxycarbamide as cytoreduction before intensive chemotherapy. Eighty-nine patients (25.7%) were admitted to the intensive care unit either during induction therapy or in the first 3 months following the first induction course. Twenty-two patients (6.3%) received an FLT3 inhibitor associated with the first induction course: four patients (1.2%) received quizartinib or placebo in the QUANTUM-FIRST clinical trial (NCT02668653), and 18 patients (5.2%) received ponatinib in the PONATINIB-AML clinical trial (NCT02428543). These patients were excluded from the efficacy and survival analyses. Among the 325 patients who received induction chemotherapy without an FLT3 inhibitor, 247 (76.0%) and 271 (83.4%) achieved CR/CRi after one or two courses, respectively, whereas 26 patients (8.0%) failed to achieve a response. Early death rate by day 30 was 8.6% (*n* = 28). Allogeneic stem cell transplantation was performed in first CR in 100 patients (36.9%).

**Figure 1:**
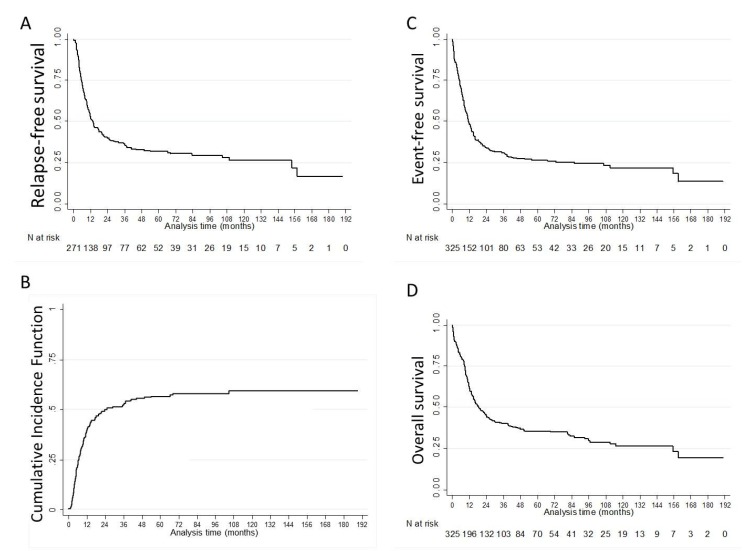
Outcome of patients with newly diagnosed *FLT3*-mutated acute myeloid leukemia: (**A**) relapse-free survival; (**B**) cumulative incidence of relapse; (**C**) event-free survival; (**D**) overall survival.

After a median follow-up of 69.9 months (IQR: 42.1–116.1), 149 out of 271 (55.0%) patients in CR/CRi relapsed. The CIR was 39.0% (95%CI: 34.0–45.0), 52.0% (95%CI: 46.0–58.0) and 57.0% (95%CI: 50.0–63.0) at 1, 3 and 5 years, respectively. The median RFS, EFS and OS were 13.6 (IQR: 5.7–154.0), 11.3 (IQR: 5.1–85.8) and 17.5 (IQR: 8.2–115.2) months, respectively (Figure 1, Table 2). Multivariate analyses showed that age ≥60 years (adjusted hazard ratio (aHR) 1.70 (95%CI: 1.29–2.24), *p* < 0.001), female gender (aHR 0.72 (95%CI: 0.54–0.94), *p* = 0.017), performance status ≥2 (aHR 1.86 (95%CI: 1.36–2.55), *p* < 0.001) and favorable cytogenetics (aHR 0.16 (95%CI: 0.04–0.65), *p* = 0.011) were significantly and independently associated with OS (Table S1). Multivariate analyses for factors associated with CR/CRi, RFS, CIR and EFS are presented in the Supplementary Data (Tables S2–S5).

### 3.3. Characteristics of Relapsed or Refractory FLT3-Mutated AML

In this series, there were 186 relapses, of which 12 (6.5%) were FLT3-wt at relapse and were therefore excluded from analyses. Thus, the total number of R/R patients was 174 (48 primary induction failure (27.6%) and 126 relapses (72.4%)). Among these 126 relapsed patients, 48 patients (27.6%) relapsed within the first 6 months after CR/CRi and 78 (44.8%) relapsed after 6 months. Among these 126 patients, 29 (23.0%) relapsed after allogeneic HSCT. The median time to relapse was 7.7 months (IQR: 4.7–12.6). Among the 48 refractory patients, 12 (25.0%) and 36 (75.0%) received one or two induction courses, respectively. The characteristics of these 174 R/R *FLT3*-mutated AML patients are shown in Table 3.

### 3.4. Treatment of Relapsed or Refractory FLT3-Mutated AML

One hundred twenty-nine patients (74.1%) received a salvage therapy: 41 (31.8%) received I/HDAC-based salvage chemotherapy; 48 (37.2%) received an anthracycline-based chemotherapy (either “7 + 3”-like or combined to I/HDAC); 10 (7.8%) received a GO-based salvage chemotherapy, 6 (4.7%) received azacitidine and 3 (2.3%) received LDAC. Five patients (3.9%) received another type of treatment (radiotherapy for extramedullary localization, dactinomycin or all-*trans* retinoic acid). Twenty-five patients (19.4%) received a FLT3 inhibitor-containing regimen; 11 patients received gilteritinib or quizartinib as a single agent, one patient received midostaurin with dactinomycin, and 13 patients received sorafenib alone or associated to dactinomycin, LDAC or all-*trans* retinoic acid. In total, 99 patients (76.7%) received high-intensity chemotherapy whereas nine patients (7.0%) received azacitidine or LDAC and 17 patients (13.2%) received low-intensity chemotherapy. Finally, four patients (3.1%) received allogeneic hematopoietic stem cell transplantation (HSCT) after sequential conditioning without previous salvage chemotherapy. Forty-five patients (25.9%) received best supportive care only (Figure S1).

The 11 patients who received gilteritinib or quizartinib as a single agent or midostaurin with dactinomycin were excluded from efficacy analyses, whereas those receiving sorafenib were included among the low-intensity regimens because this treatment was considered “as a compassionate real-world approach” in this setting. Three more patients were also excluded because of loss of follow-up. In total, 114 patients (32 refractory (28.1%) and 82 relapses (71.9%)) received salvage treatment other than quizartinib, gilteritinib and midostaurin. CR/CRi was achieved in 57 patients (50.0%). Fifty-two patients (45.6%) failed to achieve CR/CRi and the five remaining patients (4.4%) died before evaluation. Thirty-nine patients (34.2%) could proceed to allogeneic HSCT.

### 3.5. Outcome of Relapsed or Refractory FLT3-Mutated AML

**Figure 2:**
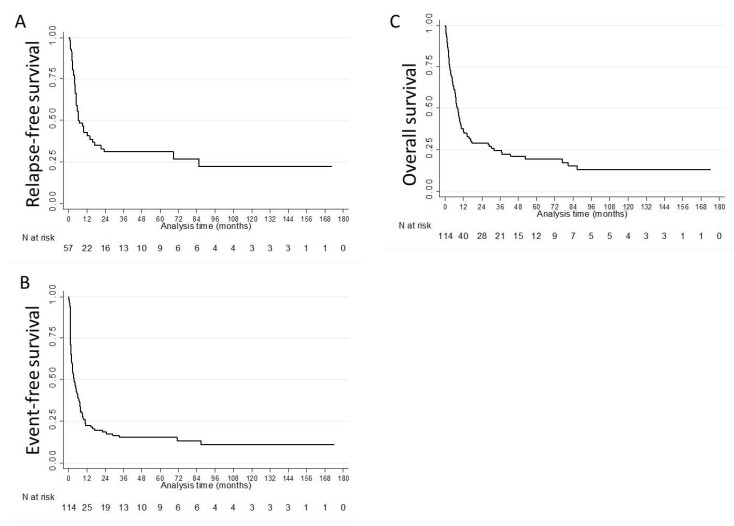
Outcome of patients with refractory or relapsed *FLT3*-mutated acute myeloid leukemia: (**A**) relapse-free survival; (**B**) event-free survival; (**C**) overall survival.

The 30-day and 60-day death rates were 6.1% (*n* = 7) and 14.9% (*n* = 17). Median duration of response in primary refractory and relapsed patients were 4.2 (IQR: 3.3–12.0) and 5.7 (IQR: 2.4–9.7) months, respectively. With a median follow-up of 63.9 months (IQR: 34.6–130.0), the median RFS, EFS and OS were 6.8 (IQR: 3.6–85.6), 3.4 (IQR: 1.3–10.6) and 8.2 (IQR: 3.0–32.0) months, respectively (Figure 2, Table 4). Multivariate analyses for factors significantly and independently associated with the various endpoints were not sufficiently powered to draw any conclusion [17]. In univariate analyses, FLT3 ratio ITD/wt (≤0.78 versus >0.78) was not significantly associated with RFS (*p* = 0.5893), EFS (*p* = 0.6131), OS (*p* = 0. 6758) or second response (*p* = 0.4160).

## 4. Discussion

Most AML patients refractory to standard induction chemotherapy or relapsing after complete response die from disease progression. A longer relapse-free interval after first CR, presence of a CBF-AML at diagnosis, lower age at relapse and no previous stem-cell transplantation during first-line therapy are factors associated with more favorable prognosis in this setting but concern a minority of patients [18]. Moreover, FLT3-ITD is an independent poor prognostic factor in R/R AML patients when treated by intensive chemotherapy at time of relapse or refractory disease [19]. This has been confirmed in two randomized phase 3 trials for FLT3-mutated AML assessing FLT3 inhibitors versus standard of care, in which results of control arms were particularly poor [8,10].

In the ADMIRAL trial for patients with R/R *FLT3*-mutated AML, single-agent gilteritinib was found to significantly improve the median OS from 5.6 to 9.3 months compared with the conventional care regimen. Here, we examined the characteristics and outcome of R/R *FLT3*-mutated AML patients included in the Toulouse–Bordeaux DATAML registry, in order to evaluate the efficacy of standard treatments that appeared to have limited efficacy in the ADMIRAL study. Our study reflects routine practice in a registry that covers a region of 6 million inhabitants (approximately 10% of the French population). One potential source of bias in the current study is *FLT3* status, which was known in 56% of all patients added to the DATAML registry between 2000 and 2017, especially in the early 2000s. However, the *FLT3* status is known in 66% of patients selected for intensive chemotherapy and, among them, for 81% of patients with intermediate cytogenetic risk. Moreover, because FLT3-ITD but not FLT3-TKD had a long-lasting prognostic value, FLT3-TKD has not been systematically monitored until recently with the advent of FLT3 inhibitors such as midostaurin, approved for first-line therapy in 2017. Therefore, FLT3-TKD patients are likely underrepresented in our study. Also, there may be measurement bias in the comparison of the remission rates based on real-world data versus a prospective trial, but this measurement bias obviously does not apply to OS or to the allogeneic HSCT rate, which are objective endpoints. The main patient characteristics were roughly similar between the salvage chemotherapy group of ADMIRAL and our cohort, apart from a slightly younger median age. This could partly explain the differences in treatment allocation, as 20.2% of patients received a low-intensity regimen as salvage treatment in our study (among them, one third received LDAC or azacitidine), whereas 39.5% of patients were allocated to LDAC or azacitidine in the ADMIRAL control arm. The rate of CR or CRi in our study was 50.0% compared to 15.3% in the standard arm of the ADMIRAL study (16% CR patients treated with high intensity chemotherapy). Moreover, the transplantation rate in our study (34.2%) was higher than in the standard arm of the ADMIRAL trial (15%). Thus, the higher response and transplantation rates may explain the better outcome observed in our cohort compared to the control arm of the ADMIRAL trial.

Obviously, our results do not mean that real-world treatments are equivalent to those of gilteritinib. For example, ADMIRAL patients preselected for high-intensity chemotherapy and randomized to receive gilteritinib had a median OS of 10.5 months, indicating that the way patients are selected for a given treatment influences outcome.

The adverse effects of salvage treatments were not specifically addressed in this study because it is well established that high-intensity regimens, including high-dose cytarabine, FLAG-IDA regimen or equivalents, are very toxic in terms of use of healthcare resources, length of hospital stay, transfusion support, infections and quality of life. In the ADMIRAL study, 30-day and 60-day mortality were 2.0% and 7.7% in the gilteritinib arm compared to 10.2% and 19.0% in the control arm or 6.1% and 14.9% in our study. Quality of life could also be improved with this oral targeted agent compared to high-intensity chemotherapy; this makes sense but is not clearly demonstrated to date.

## 5. Conclusions

Given the underperforming results of second-line therapeutic strategies, the treatment of R/R *FLT3*-mutated AML patients remains a major challenge. Our study shows that some patients could still benefit from intensive chemotherapy (i.e., achieving a CR while maintaining a suitable performance status to allow transplantation), although we failed (because of too small sample size) to identify factors that could help identify patients who will benefit. This suggests that using combinations or sequential treatments based on gilteritinib and chemotherapy could improve results. Obviously, combinations of targeted therapies such as FLT3 inhibitors and BCL2 inhibitors, for example, are also promising options for R/R *FLT3*-mutated AML patients [20].

## Figures and Tables

**Table 1:** Baseline characteristics of the 347 newly diagnosed *FLT3*-mutated AML patients treated with intensive chemotherapy.

Patients’ Characteristics	*N* = 347
Age (years)	
Median (IQR)	57.3 (47.8–67.6)
Range	18.6–81.4
Gender: *n* (%)	
Female	176 (50.7)
Male	171 (49.3)
ECOG performance status: *n* (%)	
0–1	226 (73.9)
≥2	80 (26.1)
WBC (× 109/L)	
Median (IQR)	52.6 (20.6–117.8)
Range	0.4–433.0
Tumor burden: *n* (%)	
Extramedullary involvement	
Yes	137 (42.7)
No	184 (57.3)
Leukostasis	
Yes	55 (15.5)
No	289 (84.5)
LDH	
>normal	311 (93.4)
normal	22 (6.6)
Biochemistry: median (IQR)	
Creatinine (µmol/L)	80.0 (64.0–101.0)
Albumin (g/L)	36.0 (32.0–39.5)
Fibrinogen (g/L)	4.0 (2.8–5.3)
AML status: *n* (%)	
De novo	306 (88.4)
Secondary AML	40 (11.6)
Cytogenetic risk: *n* (%)	
Favorable	13 (3.7)
Intermediate	318 (91.6)
Normal	255/311 (82.0)
Intermediate-abnormal	56/311 (18.0)
Adverse	16 (4.6)
ELN 2010 classification: *n* (%)	
Favorable	27 (8.2)
Intermediate-1	232 (70.1)
Intermediate-2	56 (16.9)
Adverse	16 (4.8)
FLT3 mutation: *n* (%)	
ITD	317/342 (92.7)
TKD	39/141 (27.7)
FLT3 ratio ITD/wt: *n* (%)	
0.03–0.25	34 (24.1)
0.26–0.50	40 (28.4)
0.51–0.78	43 (30.5)
>0.78	24 (17.0)
NPM1: *n* (%)	
Mutation	214 (65.6)
No mutation	112 (34.4)
IDH1/2 mutations: *n* (%)	
IDH1R132	13 (7.6)
IDH2R140	9 (5.3)
IDH2R172	0 (0.0)
No mutation	148 (87.1)
Induction chemotherapy	
Daunorubicin–cytarabine	127 (36.6)
Idarubicin–cytarabine	101 (29.1)
Idarubicin–cytarabine–lomustine	103 (29.7)
Daunorubicin–cytarabine–gemtuzumab ozogamicin	8 (2.3)
Other	8 (2.3)
Allogeneic stem cell transplantation in first CR: *n* (%)	100/271 (36.9)

AML: acute myeloid leukemia; CR: complete remission; ELN: European LeukemiaNet; IQR: interquartile range; ITD: internal tandem duplication; LDH: lactate dehydrogenase; TKD: tyrosine kinase domain; WBC: white blood cells count; wt: wild-type.

**Table 2:** Survival of *FLT3*-mutated acute myeloid leukemia after first-line FLT3 inhibitor-free intensive chemotherapy.

Endpoint	*N*	Median (Months, (IQR))	1-Year % (95%CI)	3-Year % (95%CI)	5-Year % (95%CI)
**RFS**	271	13.6 (5.7–154.0)	52.7 (46.5–58.5)	36.3 (30.4–42.1)	31.8 (26.1–37.7)
**CIR**	271		39.0 (34.0–45.0)	52.0 (46.0–58.0)	57.0 (50.0–63.0)
**EFS**	325	11.3 (5.1–85.8)	48.0 (42.4–53.3)	31.0 (25.9–36.2)	26.5 (21.6–31.6)
**OS**	325	17.5 (8.2–115.2)	62.0 (56.4–67.0)	40.1 (34.6–45.5)	35.5 (30.0–41.0)

CI: confidence interval; CIR: cumulative incidence of relapse; EFS: event-free survival; IQR: interquartile range; RFS: relapse-free survival; OS: overall survival.

**Table 3:** Characteristics of patients with relapsed/refractory *FLT3*-mutated acute myeloid leukemia (*n* = 174).

Patients’ Characteristics	R/R FLT3-Mutated AML *N* = 174
Age (years):	
Refractory: Median (IQR)	57.8 (43.1–67.3)
Relapse: Median (IQR)	59.9 (47.2–70.7)
Gender: *n* (%)	
Female	88 (50.6)
Male	86 (49.4)
ECOG performance status: *n* (%)	
0–1	106 (79.7)
≥2	27 (20.3)
Status: *n* (%)	
Refractory	48 (27.6)
One induction course	12 (6.9)
Two induction courses	36 (20.7)
Relapse	126 (72.4)
<6 months	48 (27.6)
≥6 months	78 (44.8)
Duration of CR/CRi before relapse (months):	
Median (IQR)	7.7 (4.7–12.6)
Previous allogeneic HSCT in first CR: *n* (%)	29 (23.0)
FLT3 ITD/wt ratio (*N* = 65) (%):	
Median (IQR)	50.0 (28.0–68.0)
Co-mutations: *n* (%)	
NPM1 mutations	
Yes	94 (56.6)
No	72 (43.4)
DNMT3A mutations	
Yes	20 (29.9)
No	47 (70.1)
CEBPA mutations	
Yes	6 (7.4)
No	75 (92.6)
IDH1/2 mutations	
Yes	8 (9.4)
No	77 (90.6)
N/K RAS mutations	
Yes	3 (8.6)
No	32 (91.4)
WBC (×109/L):	
At diagnosis (refractory)	
Median (IQR)	72.8 (18.3–149.8)
Range	0.6–317.0
At relapse	
Median (IQR)	7.4 (3.4–26.9)
Range	0.1–436.0

CR: complete remission; CRi: CR with incomplete hematological recovery; HSCT: hematologic stem cell transplantation; IQR: interquartile range; WBC: white blood cells count; wt: wild-type.

**Table 4:** Survival of relapsed/refractory FLT3-mutated AML after salvage treatment.

	N	Median (Months, (IQR))	1-Year % (95%CI)	3-Year % (95%CI)	5-Year % (95%CI)
**RFS**	57	6.8 (3.6–85.6)	42.9 (29.8–55.3)	31.2 (19.4–43.7)	31.2 (19.4–43.7)
**EFS**	114	3.4 (1.3–10.6)	22.4 (15.2–30.4)	15.4 (9.3–22.9)	15.4 (9.3–22.9)
**OS**	114	8.2 (3.0–32.0)	36.0 (27.2–44.8)	24.7 (17.0–33.3)	19.7 (12.5–28.2)

CI: confidence interval; CIR: cumulative incidence of relapse; EFS: event-free survival; IQR: interquartile range; RFS: relapse-free survival; OS: overall survival.

## Supplementary Materials

The following are available online at https://www.mdpi.com/2072-6694/12/4/773/s1, Figure S1: Flowchart, Table S1: Cox model for factors independently associated with overall survival among *FLT3*-mutated AML patients, Table S2: Logistic regression model for factors independently associated with CR/CRi among *FLT3*-mutated AML patients, Table S3: Cox model for factors independently associated with relapse-free survival among *FLT3*-mutated AML patients, Table S4: Cox model for factors independently associated with cumulative incidence of relapse among FLT3-mutated AML patients, Table S5: Cox model for factors independently associated with event-free survival among *FLT3*-mutated AML patients.
