# Supplementary material for: Outcome of Relapsed or Refractory FLT3-Mutated Acute Myeloid Leukemia before Second-Generation FLT3 Tyrosine Kinase Inhibitors: A Toulouse–Bordeaux DATAML Registry Study
Source: Cancers (Basel). 2020 Mar 25;12(4):773. doi: 10.3390/cancers12040773 (PMC7226007; doi:10.3390/cancers12040773)
Supplement: Supplementary file 1 [file cancers-12-00773-s001.pdf]

Supplementary Materials

Outcome of Relapsed or Refractory *FLT3*-Mutated Acute Myeloid Leukemia Before Second-Generation FLT3 Tyrosine Kinase Inhibitors: a Toulouse–Bordeaux DATAML Registry Study

Sarah Bertoli, Pierre-Yves Dumas, Emilie Bérard, Laetitia Largeaud, Audrey Bidet, Eric Delabesse, Suzanne Tavitian, Noémie Gadaud, Thibaut Leguay, Harmony Leroy, Jean-Baptiste Rieu, Jean-Philippe Vial, François Vergez, Nicolas Lechevalier, Isabelle Luquet, Emilie Klein, Audrey Sarry, Anne-Charlotte De Grande, Christian Récher and Arnaud Pigneux

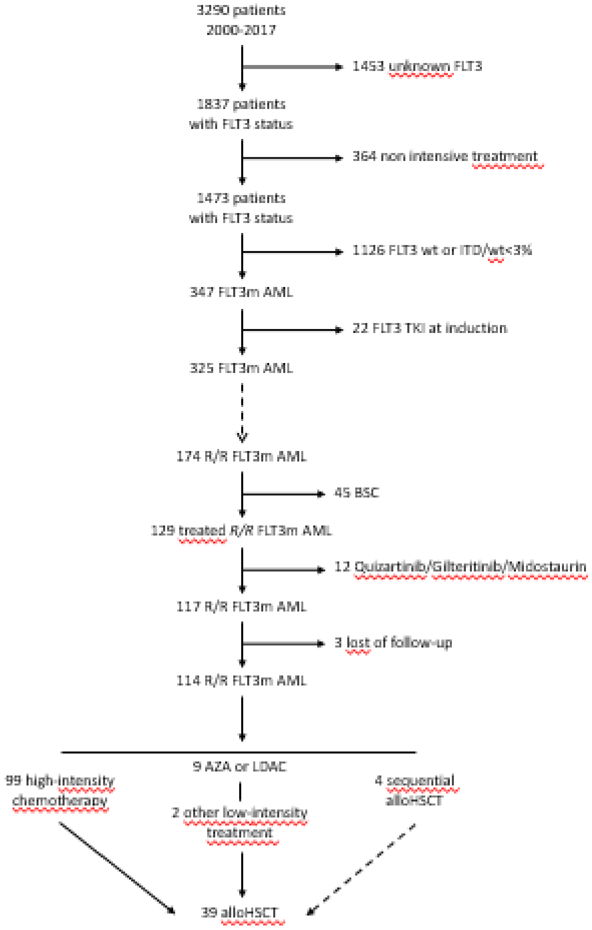

Figure S1. Flowchart.

Table S1. Cox model for factors independently associated with overall survival among *FLT3*-mutated AML patients.

	N	Events	aHR	95%CI	P-value
**Age subgroups**					
< 60 years	185	106	1	-	-
≥ 60 years	140	105	1.70	1.29-2.24	<0.001
**Gender**					

Male	162	115	1	-	-
Female	163	96	0.72	0.54-0.94	0.017
**ECOG at diagnosis**					
0-1	210	120	1	-	-
≥ 2	76	58	1.86	1.36-2.55	<0.001
**Cytogenetic risk**					
Intermediate	298	200	1	-	-
Favorable	13	2	0.16	0.04-0.65	0.011
Adverse	14	9	0.95	0.48-1.85	0.869

aHR: adjusted hazard ratio, CI: confidence interval, ECOG: *performance status.*

Table S2. Logistic regression model for factors independently associated with CR/CRi among *FLT3*-mutated AML patients.

	N	Events	aOR	95%CI	P-value
**Age subgroups**					
< 60 years	185	166	1	-	-
≥ 60 years	140	105	0.39	0.20-0.74	0.004
**ECOG at diagnosis**					
0-1	210	190	1	-	-
≥ 2	76	57	0.41	0.20-0.84	0.015
**WBC at diagnosis**					
< 50 x10^9/L	151	139	1	-	-
≥ 50 x10^9/L	174	132	0.42	0.20-0.87	0.019

aOR: adjusted odds ratio, CI: confidence interval, ECOG: *performance status,* WBC: white blood cells.

Table S3. Cox model for factors independently associated with relapse-free survival among *FLT3*-mutated AML patients.

	N	Events	aHR	95%CI	P-value
**Age subgroups**					
< 60 years	166	103	1	-	-
≥ 60 years	105	80	1.44	1.07-1.94	0.015
**ECOG at diagnosis**					
0-1	190	121	1	-	-
≥ 2	57	43	1.48	1.04-2.10	0.029
**Cytogenetic risk**					
Intermediate	244	169	1	-	-
Favorable	13	4	0.34	0.13-0.93	0.035
Adverse	14	10	1.20	0.63-2.29	0.573

aHR: adjusted hazard ratio, CI: confidence interval.

Table S4. Cox model for factors independently associated with cumulative incidence of relapse among *FLT3*-mutated AML patients.

	N	Events	sHR	95%CI	P-value
**Cytogenetic risk**					
Intermediate	244	135	1	-	-
Favorable	13	4	0.40	0.16-1.01	0.051
Adverse	14	10	1.99	1.07-3.72	0.031
**Allogeneic HSCT**					
No	171	115	1	-	-

Yes	100	34	0.43	0.28-0.64	< 0.001
**NPM1 co-mutation**					
No	85	54	1	-	-
Yes	169	89	0.68	0.48-0.95	0.025

sHR: subhazard ratio, CI: confidence interval, HSCT: hematopoietic stem cell transplantation (as time-dependent covariate).

Table S5. Cox model for factors independently associated with event-free survival among *FLT3*-mutated AML patients.

	**N**	**Events**	**HR**	**95%CI**	**P-value**
**ECOG at diagnosis**					
0-1	210	141	1	-	-
≥ 2	76	62	1.40	1.03-1.91	0.030
**Cytogenetic risk**					
Intermediate	298	223	1	-	-
Favorable	13	4	0.31	0.11-0.82	0.019
Adverse	14	10	1.22	0.64-2.31	0.553
**Allogeneic HSCT**					
No	171	128	1	-	-
Yes	100	55	0.69	0.49-0.95	0.025

HR: hazard ratio, CI: confidence interval, ECOG: *performance status.*

© 2020 by the authors. Licensee MDPI, Basel, Switzerland. This article is an open access article distributed under the terms and conditions of the Creative Commons Attribution (CC BY) license (http://creativecommons.org/licenses/by/4.0/).
